# Supplementary material for: Interactions between an Associative Amphiphilic Block Polyelectrolyte and Surfactants in Water: Effect of Charge Type on Solution Properties and Aggregation
Source: Polymers (Basel). 2021 May 25;13(11):1729. doi: 10.3390/polym13111729 (PMC8197838; doi:10.3390/polym13111729)
Supplement: Supplementary file 1 [file polymers-13-01729-s001.zip › polymers-1238404-supplementary.pdf]

polymers

MDPI

Supplementary Materials

Interactions Between an Associative Amphiphilic Block Polyelectrolyte and Surfactants in Water: Effect of Charge Type on Solution Properties and Aggregation

Patrizio Raffa *

Department of Chemical Engineering, Faculty of Science and Engineering, University of Groningen. Nijenborgh 4, 9747 AG, Groningen, the Netherlands
* Correspondence: p.raffa@rug.nl

Surfactants characterization

Surface tension curves for the studied surfactants are reported in Figure S1. Except for PEGMe, that has low hydrophobicity, they all present a clear CMC. The nonionic surfactants differ by their surface activity (Figure S1, supporting information). Moreover, they can be considered polymeric surfactants, due to their relatively high molecular weight. These are usually interesting from a point of view of rheological properties.[1]

The values for Pluronic [2] and CTAB [3] measured here are in agreement with values reported in literature.

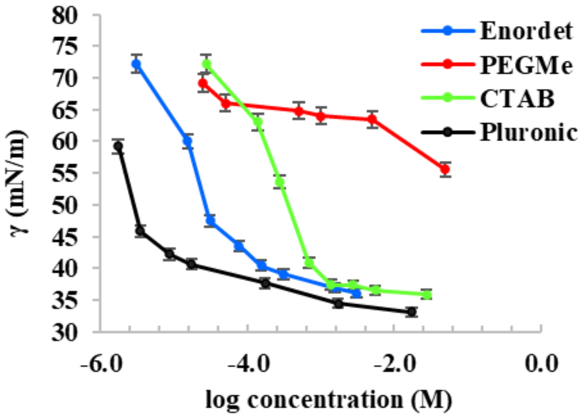

Figure S1. Surface tension curves of the studied surfactants.

Polymer characterization.

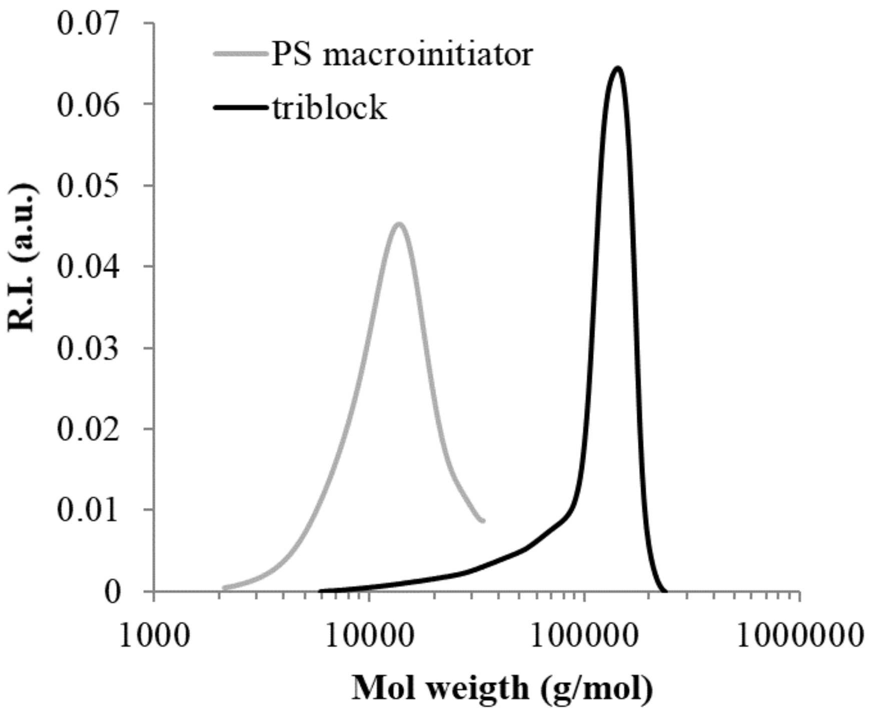

Figure S2. GPC traces in THF of the triblock copolymer studied in this work (**black line**) before hydrolysis, and polystyrene macroinitiator (**grey line**). After hydrolysis, GPC is not possible due to aggregation of the polymer. The full characterization of the polymer (including GPC conditions) has been previously reported.[4].

Surface tension measurements

Surface tension plots discussed in section 3.1.2 and 3.1.3 are reported here below

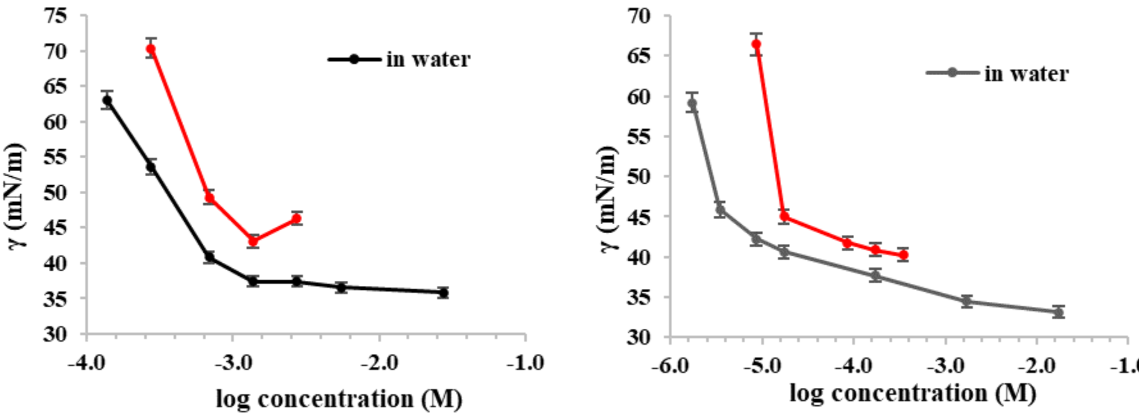

Figure S3. Surface tension curve of CTAB (**left**) and Pluronic (**right**) in absence and presence of 0.5 wt % polymer.

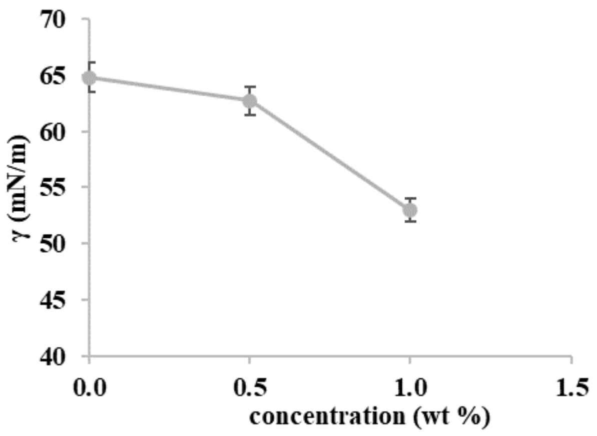

Figure S4. Surface tension values of 0.1 wt % solution of PEGMe at different polymer concentrations.

Rheological measurements

Shear viscosity plots discussed in section 3.2 are reported here below

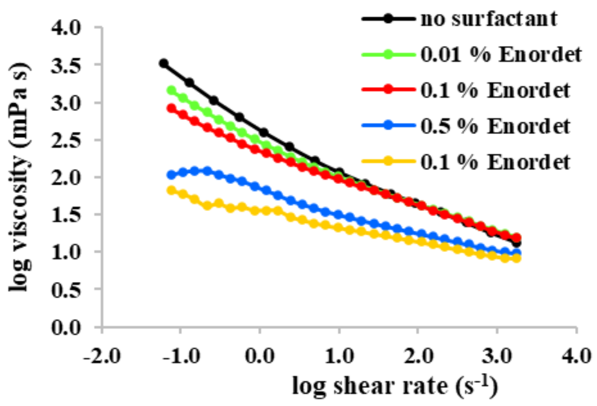

Figure S5. Shear viscosity of a 0.1 wt % polymer solution with increasing amount of Enordet.

References

1. 1. Raffa, P.; Wever, D.A.Z.; Picchioni, F.; Broekhuis, A.A. Polymeric Surfactants: Synthesis, Properties, and Links to Applications. *Chem. Rev.* **2015**, *115*, 8504–8563, doi:10.1021/cr500129h.
2. 2. Alexandridis, P.; Holzwarth, J.F.; Hatton, T.A. Micellization of Poly(ethylene oxide)-Poly(propylene oxide)-Poly(ethylene oxide) Triblock Copolymers in Aqueous Solutions: Thermodynamics of Copolymer Association. *Macromol.* **1994**, *27*, 2414–2425, doi:10.1021/ma00087a009.
3. 3. Ekwall, P.; Mandell, L.; Solyom, P. The aqueous cetyl trimethylammonium bromide solutions. *J. Colloid Interface Sci.* **1971**, *35*, 519–528, doi:10.1016/0021-9797(71)90210-4.
4. 4. Raffa, P.; Brandenburg, P.; Wever, D.A.Z.; Broekhuis, A.A.; Picchioni, F. Polystyrene–Poly(sodium methacrylate) Amphiphilic Block Copolymers by ATRP: Effect of Structure, pH, and Ionic Strength on Rheology of Aqueous Solutions. *Macromol.* **2013**, *46*, 7106–7111, doi:10.1021/ma401453j.
